# Impact after 3 years of application of enteral paromomycin to eradicate colistin and carbepemenase resistant microrganisms in rectal colonization to prevent ICU infections

**DOI:** 10.1186/2197-425X-3-S1-A130

**Published:** 2015-10-01

**Authors:** C Sánchez Ramirez, L Caipe Balcázar, MA Hernández Viera, M Cabrera Santana, S Hípola Escalada, N Sangil Monroy, F Artiles Campelo, CF Lübbe Vazquez, MA De la Cal Lòpez, S Ruiz Santana

**Affiliations:** University Hospital of Gran Canaria Dr Negrín, Intensive Care Unit, Las Palmas de Gran Canaria, Spain; Microbiology Department, Las Palmas de Gran Canaria, University Hospital of Gran Canaria Dr Negrín, Spain; University Hospital of Getafe, Intensive Care Unit, Madrid, Spain

## Introduction

Emergence of carbapemenases and their worldwide distribution has worsens the clinical scenario of antibiotic resistance in Gram-negative bacteria. Selective Digestive Decontamination (SDD) has been used to prevent development of nosocomial colonization and infections in ICU patients. However, rectal colonization with multirresistant bacteria is a serious concern with SDD. An effective enteral antimicrobial treatment in this scenario is still lacking and it might contribute to prevent systemic nosocomial infections.

## Objective

To assess the value of enteral paromomycin to decontaminate patients with rectal colistin and / or carbepemenase resistant microorganisms colonization to prevent the development of ICU nosocomial infections.

## Methods

All consecutive patients admitted to the ICU from October 2011 to February 2015, expected to require tracheal intubation for longer than 48 hours were given SDD with a 4-day course of intravenous cefotaxime, plus enteral colistin, tobramycin, nystatin in an oropharyngeal paste and in a digestive solution. Oropharyngeal and rectal swabs were obtained on admission and once weekly. Patients with rectal swabs colonized by colistin and / or carbepemenase resistant microorganisms were treated with enteral paromomycin 1 gr every 6 hours daily, in order to negativize it and eventually preventing nosocomial infections. Categorical variables were summarized as frequencies and percentages and the continuous ones as medians and interquartile ranges (IQR) or means and standard deviations. Statistical significance was set at *p* ≤ 0.05.

## Results

**Figure 1:**
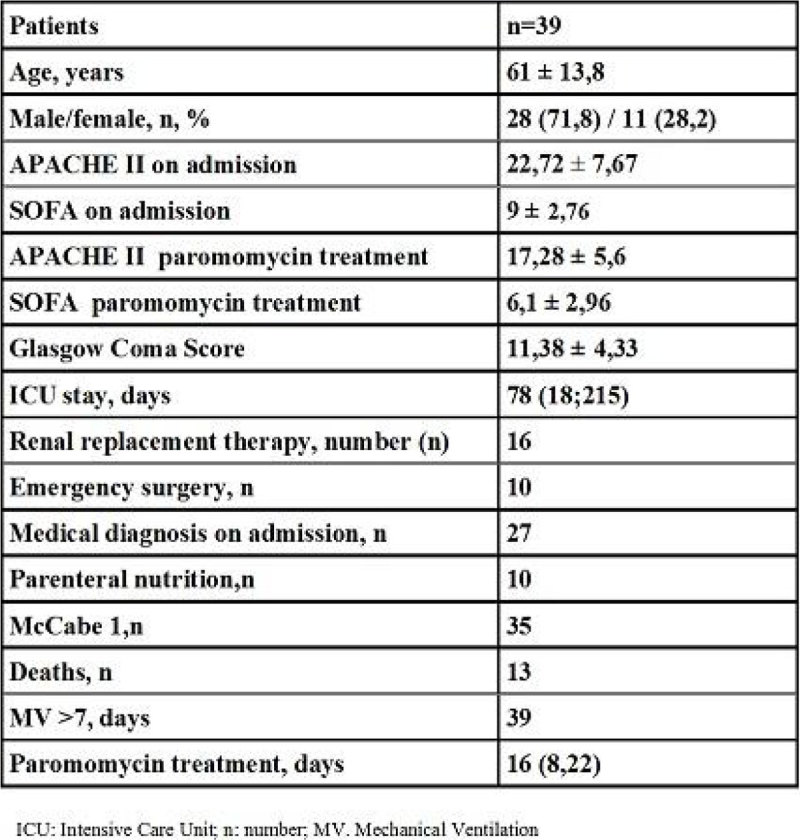
**Patients data.**

We applied paromomycin treatment to 39 patients colonized with rectal colistin resistant microorganisms. All of them had colonization by Extended Spectrum Beta-lactamases (ESBLs) *Klebsiella pneumoniae.* Demographic data and type of admission are shown in Figure 1.

Twenty nine (74.3%) out of 39 of the studied patients negativized the rectal swab. Three of them (7.6%) were colonized by carbepemenases producing microorganisms and only one of them died with persistent multirresistant rectal colonization. Only 9 out of the 29 patients that negativized the colonization received concurrent susceptible IV antibiotics. None of the patients treated with paromomycin developed any infection including ESBL *Klebsiella pneumoniae*. Finally, 13 patients died in the ICU.

## Conclusions

Our preliminary data show that enteral paromomycin is useful in treating rectal colistin and / or carbepemenase resistant microorganisms colonization to prevent the development of ICU nosocomial infections.
